# Supplementary material for: Distribution Characteristics and Risk Assessment of Agricultural Land Use Non-Point Source Pollution in Typical Biofuel Ethanol Planting Areas
Source: Int J Environ Res Public Health. 2022 Jan 26;19(3):1394. doi: 10.3390/ijerph19031394 (PMC8835376; doi:10.3390/ijerph19031394)
Supplement: Supplementary file 1 [file ijerph-19-01394-s001.zip › ijerph-1483174-supplementary.pdf]

Table S1 Nitrogen and phosphorus indicators of soil sample points (maize planting area) mg kg^{-1}

Points	Crops	TN	NO^{3-}-N	NH_4^+-N	TP
1	maize	1730	23.650	1.317	272.00
2	maize	380	19.763	2.962	485.00
3	maize	1290	6.260	49.704	504.00
4	rice	3120	6.230	6.131	505.00
5	maize	960	23.210	15.131	545.00
6	maize	2525	11.231	31.131	510.00
10	maize	1653	6.053	7.376	740.00
11	maize	1540	5.391	7.590	447.00
12	maize	1913	6.996	13.636	819.00
18	maize	1720	7.373	13.293	579.00
19	maize	2090	9.611	10.849	617.00
20	maize	1640	5.391	9.305	801.00
21	soybeans	1300	8.316	3.688	651.00
22	maize	1540	8.128	6.518	556.00
23	maize	2570	7.265	12.822	851.00
24	maize	3100	5.768	4.974	362.00
25	maize	1990	6.542	11.278	541.00
30	maize	740	7.885	4.245	766.00
31	maize	1620	10.013	11.364	390.00
32	maize	1840	5.774	4.674	620.00
33	maize	2460	12.297	6.775	740.00
34	maize	2100	10.570	5.403	700.00
35	vegetable	2100	10.762	5.575	257.00
36	forest	1590	12.276	5.146	730.00
37	rice	1080	3.856	7.333	525.00
38	forest	2950	69.082	3.431	875.00
39	maize	2070	6.242	15.352	573.00
40	maize	1730	4.432	19.383	607.00
41	soybeans	2340	16.326	3.816	225.00
42	forest	1820	10.570	8.190	247.00
43	forest	1480	5.391	5.875	637.52
44	maize	2100	74.261	85.720	772.50
45	forest	1510	6.734	8.877	669.58
46	maize	3980	10.013	9.605	411.74
47	maize	2360	9.259	5.832	406.60
48	maize	2190	10.391	11.835	104.34
49	maize	1500	10.202	3.988	865.27
50	maize	3740	10.202	13.679	664.72
51	maize	1840	10.013	7.419	202.05
52	maize	1810	11.146	3.559	624.55

53	maize	1920	11.334	9.820	566.45
54	maize	3140	29.946	7.976	728.63
55	maize	1770	8.882	6.990	592.61
56	soybeans	2070	9.071	51.350	676.76
57	maize	1833	11.711	11.621	537.08
58	unused	1220	7.751	7.333	230.78
59	maize	2030	8.693	4.588	893.43
60	maize	1690	8.882	4.674	494.20
61	maize	3690	8.505	5.703	422.97
62	maize	1520	7.185	5.017	862.96
63	maize	1600	10.663	8.748	703.93
64	soybeans	2360	15.771	6.518	606.61
65	maize	2100	9.811	7.504	968.35
66	maize	2420	7.896	17.238	172.42
67	rice	3670	6.406	6.861	325.39
68	maize	1910	8.882	22.470	187.14
69	maize	1395	8.693	6.604	191.34
70	maize	1670	14.162	7.461	5.26
71	maize	2800	18.628	4.288	215.00
72	maize	2610	11.146	5.832	873.46
73	rice	1630	5.966	3.516	509.08
74	maize	2010	4.916	6.518	402.76
75	maize	1615	7.283	7.242	505.00
76	maize	1160	6.465	8.319	545.00
77	maize	1450	13.216	19.092	510.00
78	maize	1215	8.101	49.375	740.00
79	maize	650	6.056	29.954	447.00
80	maize	2240	7.692	79.658	819.00
81	maize	2065	7.488	44.108	579.00
82	maize	1850	4.010	17.775	617.00
83	maize	1630	6.122	10.334	801.00
84	maize	1720	37.971	6.583	801.00
85	maize	1650	9.386	6.218	556.00
86	maize	2210	5.391	14.194	851.00
87	maize	2190	16.215	47.400	651.00
88	maize	2160	8.747	8.533	541.00
89	maize	990	12.807	0.658	556.00
90	maize	1270	7.692	3.621	851.00
91	maize	300	12.398	5.267	362.00
92	maize	1105	4.419	4.279	541.00
93	soybeans	2265	6.120	14.812	766.00
94	maize	1915	16.899	12.837	390.00
95	maize	1770	11.170	21.066	1170.00

96	maize	2090	3.120	4.279	740.00
97	maize	1820	15.160	4.974	700.00
98	maize	210	28.355	1.975	257.00
99	maize	2180	15.558	6.389	607.00
100	maize	2380	18.325	5.103	225.00
101	maize	2700	28.540	5.832	247.00
102	maize	2380	18.325	9.906	411.79
103	maize	2330	87.068	13.894	400.74
104	maize	2130	7.257	6.861	453.61
105	maize	3820	30.243	13.250	590.00
106	maize	2900	11.088	6.518	265.74
107	maize	1760	33.861	13.208	726.29
108	maize	2270	24.497	0.257	379.84
109	maize	2520	15.132	8.362	786.96

Table S2 Nitrogen and phosphorus indicators of soil sample points (cassava planting area) mg kg^{-1}

Points	Crops	TN	NO^{3-}-N	NH_4^+-N	TP
1	maize	1620	0.410	5.21012	625.40
2	cassava	1610	0.140	15.20154	148.97
3	rice	2700	0.336	6.625214	413.29
4	rice	2980	0.221	6.177	464.28
5	cassava	3030	0.160	3.880789	310.62
6	maize	1730	0.401	4.566895	217.51
7	maize	720	0.258	7.161	287.70
8	cassava	310	0.111	4.309605	264.40
9	cassava	870	0.156	7.783019	377.21
10	cassava	1330	0.426	6.818	549.77
11	cassava	1400	0.352	8.062	675.13
12	maize	1050	1.956	7.268439	196.68
13	cassava	1270	0.970	12.907	549.29
14	maize	980	0.504	5.295883	252.82
15	maize	1070	1.396	9.434	233.92
16	rice	1330	0.242	1.993997	141.73
17	cassava	1190	0.119	3.816	196.32
18	sugarcane	3240	0.164	3.431	265.95
19	cassava	1060	0.123	18.696	293.90
20	cassava	1800	2.533	6.261	763.54
21	rice	3300	0.332	3.023156	598.83
22	maize	1340	1.322	4.524014	1061.28
23	rice	2480	0.160	8.705	430.09

24	soybeans	1760	0.365	6.690	290.75
25	maize	460	0.512	3.859	445.23
26	rice	2430	0.475	11.750	461.15
27	maize	1920	0.573	30.103	455.25
28	rice	2050	0.311	4.824185	448.15
29	maize	2580	1.093	4.266724	104.10
30	maize	650	4.129	3.795026	475.11
31	rice	1960	0.140	8.834	747.53
32	maize	1130	0.246	23.4777	1150.96
33	cassava	1870	0.005	4.717	146.51
34	maize	2460	1.392	8.148	818.26
35	unused	2460	0.127	5.789	106.41
36	cassava	230	0.074	2.358	349.06
37	rice	610	0.111	7.397084	427.02
38	rice	2500	0.078	8.148	858.81
39	cassava	1210	0.140	3.023156	500.41
40	maize	1230	0.164	3.237564	357.06
41	forest	600	0.054	5.98199	71.02
42	cassava	850	0.082	5.553173	253.65
43	maize	1230	2.030	16.74528	811.40
44	cassava	880	0.123	5.381647	208.70
45	unused	730	0.099	3.237564	141.28
46	cassava	1350	0.148	7.011149	409.03
47	rice	3330	0.221	5.381647	377.00
48	soybeans	1550	1.506	1.82247	382.45
49	maize	2400	1.334	2.465695	974.32
50	peanut	3190	0.811	5.124357	721.43
51	maize	710	0.573	5.7247	221.24
52	cassava	1890	0.189	5.510292	273.43
53	cassava	850	2.599	3.237564	314.43
